# Uncovering a Genetic Polymorphism Located in Huntingtin Associated Protein 1 in Modulation of Central Pain Sensitization Signaling Pathways

**DOI:** 10.3389/fnins.2022.807773

**Published:** 2022-06-28

**Authors:** Yvonne Gloor, Alain Matthey, Komla Sobo, Médéric Mouterde, Eva Kosek, Gisèle Pickering, Estella S. Poloni, Christine Cedraschi, Georg Ehret, Jules A. Desmeules

**Affiliations:** ^1^Division of Clinical Pharmacology and Toxicology, Department of Anesthesiology, Pharmacology, Intensive Care and Emergency Medicine, Geneva University Hospitals (HUG), Geneva, Switzerland; ^2^Clinical Investigation Unit, Clinical Research Center (CRC), Geneva University Hospitals (HUG), Geneva, Switzerland; ^3^Division of Cardiology, Department of Specialties of Internal Medicine, Geneva University Hospitals (HUG), Geneva, Switzerland; ^4^Anthropology Unit, Department of Genetics and Evolution, University of Geneva, Geneva, Switzerland; ^5^Department of Clinical Neurosciences, Karolinska Institutet, Stockholm, Sweden; ^6^Clinical Investigation Center, Inserm 1405, Centre Hospitalier Universitaire, Clermont-Ferrand, France; ^7^Clinical Investigation Center, Inserm 1405, University Hospital, Clermont-Ferrand, France; ^8^Institute of Genetics and Genomics of Geneva (iGE3), Geneva, Switzerland

**Keywords:** central sensitization, nociceptive flection reflex (NFR) threshold, fibromyalgia, GWAS, *HAP1*

## Abstract

Fibromyalgia syndrome (FMS) is characterized by widespread pain and increased sensitivity to nociceptive stimulus or tenderness. While familial aggregation could suggest a potential hereditary component in FMS development, isolation of genetic determinants has proven difficult due to the multi-factorial nature and complexity of the syndrome. Central sensitization is thought to be one of the key mechanisms leading to FMS in a subset of patients. Enhanced central pain signaling can be measured using the Nociceptive Flexion Reflex (NFR) or RIII threshold. We performed a genome-wide association study (GWAS) using an array to genotype 258,756 human genetic polymorphisms in 225 FMS patients and 77 healthy volunteers and searched for genetic variants associated with a lowered NFR threshold. We have identified a potential association between a single nucleotide polymorphism resulting in a common non-synonymous coding mutation in the Huntingtin associated protein 1 (*HAP1*) gene (rs4796604, MAF = 0.5) and the NFR threshold (*p* = 4.78E−06). The Hap1 protein is involved in trafficking and is particularly enriched in neurons. Our results suggest a possible involvement of the neuronal trafficking protein HAP1 in modulating pain signaling pathways and thus participate in the establishment of the NFR threshold.

## Introduction

According to the American College of Rheumatology (ACR), the fibromyalgia syndrome (FMS) is defined as a chronic pain condition of musculoskeletal origin but uncertain cause (ACR, 2022). It is a condition characterized by widespread pain and tenderness generally associated with fatigue, sleep disturbance, mood disorders, and cognitive symptoms (ACR, 2022). The first generally accepted diagnostic criteria of fibromyalgia were published only in 1990 (Wolfe et al., 1990), despite the high prevalence of the disease affecting 0.5–5% of the European (Branco et al., 2010) and 2–8% of the United States (Clauw, 2014) population. A wide range of serious co-morbidities, including depression, anxiety, and sleep disorders are frequently associated with fibromyalgia, increasing the utilization of health resources and costs (Arnold et al., 2006; Skaer, 2014).

Although the pathophysiological mechanisms leading to FMS are still poorly understood, both neuronal plasticity and central sensitization are important processes involved in the onset of FMS. Functional magnetic resonance imaging (fMRI) comparing brain activity in FMS patients versus controls revealed an increased neuronal activity in pain-processing brain-regions as well as a diminution in the connections between those regions and the anti-nociceptive regions of the brain (Sluka and Clauw, 2016). Those observations suggest that sensitization of the central nervous system (CNS) results from a physiologic acquired imbalance between incoming pain stimulation and pain inhibitory feedback loops originating from the CNS (Latremoliere and Woolf, 2009). In line with this current working model, the most potent FMS treatments available today are directed at restoring the feedback inhibitory loop with serotonin-norepinephrine reuptake inhibitors (SNRIs) or decreasing excitatory signals (like NMDA glutamate receptor antagonists) (Sluka and Clauw, 2016).

A clear familial aggregation has been observed between FMS patients (Pellegrino et al., 1989; Buskila et al., 1996; Arnold et al., 2004, 2013), with a recent study estimating a sibling recurrence risk ratio reaching 13.6 (Arnold et al., 2013). This aggregation is not observed in rheumatoid arthritis patients under similar conditions (Arnold et al., 2004) and could suggest a possible genetic background to the condition. Moreover, heritability of chronic widespread pain (CWP), which includes FMS, has been estimated to reach 30–55% depending on the study (Kato et al., 2006; Rahman et al., 2021). Although candidate gene approaches focusing on single nucleotide polymorphisms (SNPs) affecting relevant neuro-transmitters activity did uncover some polymorphisms with significantly altered distribution in FMS patients versus controls (Ablin and Buskila, 2015), most genome-wide approaches of FMS underscored the difficulty of isolating single genetic determinants of FMS (Docampo et al., 2014; Ablin and Buskila, 2015).

The Nociceptive Flexion Reflex (NFR) or R-III threshold is triggered by electrical stimulations of variable intensity applied directly to the sural nerve. It is considered as an objective and reliable assessment of central pain pathways since direct stimulation of the nerve bypasses the activation of peripheral nociceptors (Willer, 1990; Sandrini et al., 2005). The correlation between decreased NFR intensities and increased pain sensitivity is well established both in healthy volunteers and chronic pain patients (Willer, 1977; Skljarevski and Ramadan, 2002). Previous studies have shown a decrease in NFR threshold in FMS patients as compared to non-FMS control subjects (Desmeules et al., 2003; Banic et al., 2004; Tanwar et al., 2019).

Although all patients with FMS diagnosis suffer from a chronic pain condition, they represent a heterogenous group with different symptom presentation and one of the current challenges in the field concerns the sub-categorization of FMS patients in the most appropriate therapeutic group. In the current study, we used a genome-wide association study (GWAS) approach to identify genetic determinants of central sensitization using the NFR threshold as objective marker of increased sensitivity in both patients and healthy controls. We uncovered an association between a SNP in the N-terminus of the Huntingtin associated protein 1 (*HAP1*) gene and NFR threshold levels.

## Materials and Methods

### Study Population

All data and genetic material were collected during three subsequent clinical trials with patients suffering from FMS conducted by the Division of Clinical Pharmacology and Toxicology of the University Hospital of Geneva (HUG) between 1998 and 2009. The first study (AquaFM) is a case-control trial evaluating different pain assessment tools (including NFR) between FMS patients and a matched control population (Desmeules et al., 2003; Cedraschi et al., 2004). This study was followed by another case-control clinical trial (“PNR”) assessing the psychological and physiological differences between FMS patients and controls in function of the COMTVal158 genotype (Desmeules et al., 2014). Finally, the Milna study is a placebo-controlled trial investigating the pharmaco-dynamic activity of milnacipran in a cohort of FMS patients (Matthey et al., 2013). Each study was approved by the Geneva Ethics Committee (CCER 98-63, 05-052, 05-215), which also approved the reutilization of the collected biological material for the GWAS analysis (CCER 2016-01811). All clinical studies were conducted in accordance with the ethical principles of the Helsinki declaration, the ICH guidelines and Swiss national law governing clinical trials. All participants signed an inform consent form and were free of disease or medical disorder requiring immediate treatment at the time of their enrollment and during the time of the study. All FMS patients were diagnosed according to the 1990 ACR criteria based on tender point examination only. Additional symptoms such as fatigue, sleep disturbance, mood disorders, or cognitive symptoms were integrated in the diagnosis of FMS at a later time point and not considered in this study (Hauser et al., 2017).

### Nociceptive Flexion Reflex Measurements

The nociceptive flexion R-III reflex (NFR) was measured as previously described (Desmeules et al., 2003). Briefly, the sural nerve was electrically stimulated with single impulses (0.5 ms) delivered at 6–10 s intervals by a constant current stimulator with variable intensities (1–100 mA). Electromyographic responses were recorded using a pair of surface electrodes placed over the ipsilateral biceps femoris. The NFR response was identified as a multiphasic signal appearing 90–200 ms after stimulation with an AUC greater than 0.150 mV/ms. The NFR threshold (mA) was defined as the stimulation intensity generating 50% positive responses in a series of 30–40 stimulations using Hill’s equation. An upper threshold for input current intensities was set at 100 mA. Only baseline NFR thresholds measured before any study intervention, but after treatment interruption for patients able to stop their medication were considered.

### DNA Samples Preparation

DNA purification and concentration of 302 samples (225 FMS patients and 77 controls) was performed in the Laboratory for Clinical Pharmacology and Toxicology of the Geneva University Hospital (HUG) using the QIAamp DNA blood mini kit (QIAGEN). Quantification was performed with the Qubit dsDNA BR Assay (Thermo Fischer Scientific).

### Genotyping

Genotyping was performed at the iGE3 genomic platform of the University of Geneva (UniGE) using an Infinium CoreExome-24 BeadChip from Illumina containing 555,356 markers including 4000 custom markers (Mouterde et al., under review^1^), among which 906 SNPs selected on the basis of their potential association to FMS and/or central pain sensitization through literature-mining (list of FMS related SNPs in Supplementary Data 1).

Genotype intensities were called using Illumina GenomeStudio™. All sample call rates (CRs) from the genotyping experiment exceeded 99%. The mean genotyping CR for the 302 samples was 99.73%. All SNPs were evaluated by the Illumina cluster quality score and SNPs with GenTrain score < 0.7 were discarded.

In the final data set, SNPs with a genotyping CR below 98%, a significant deviation from Hardy–Weinberg equilibrium (HWE) or a minor allele frequency (MAF) below 5% (CR < 98%, HWE *p* < 10^–5^, MAF < 0.05) were discarded. All genetic coordinates refer to the Ensembl GRCh37 reference assembly.

### Genome-Wide Association Study Quality Control and Bioinformatics Analysis

Quality control and genome-wide association analysis were performed using the PLINK 1.07v software (Purcell et al., 2007). Principal component analyses (PCAs) were performed in R version 3.5.3 using the Adegenet package (v2.1.1) (Jombart, 2008; Jombart and Ahmed, 2011). Manhattan and QQ-plots were drawn using the qqman R package (v0.1.4) (Turner, 2014). Lambda statistics was calculated in R version 3.5.3.

Quality control considered relatedness, gender attribution, heterozygosity and inbreeding, genotype consistency versus HapMap phase III population of European descend, allele frequency consistency and ethnicity versus the HRC r1.1 (Human Reference Consortium version r1.1 2016) EUR population data, as well as sample reproducibility (Supplementary Data 2). A final neighborhood analysis confirmed that all outliers had been detected (Guo et al., 2014). From the initial population of 302 participants, 3 were excluded due to first-degree family relationships and 15 based on ethnicity.

Following quality control, 258,756 SNPs (46.59%) in 284 samples including 212 FMS patients and 72 controls were cleared for further analysis. The genotyping rate in the final population is >0.999.

Genome-wide association study imputation of chromosome 17 was performed using the Michigan imputation server (Minimac4) and the EUR population of the HRC r1.1 as reference panel (*r*^2^ filter = 0.3, phasing with Eagle v2.4). Following imputation, the dataset on chromosome 17 reached 348,128 SNP, which after QC filtration (MAF > 0.05, CR > 0.98, HWE > 1E−05 and imputation *R*^2^ > 0.8) left 114,238 SNPs for association analysis (versus 7100 before imputation).

Single nucleotide polymorphisms were annotated with the Annovar software using the hg19 build (Wang et al., 2010). Linkage disequilibrium (LD) analysis was performed using the Ensembl tool LD Calculator for the GRCh37 assembly and association results filtered for LD *r*^2^ < 0.3 (Hunt et al., 2018). Regional association plots for chromosome 17 were prepared using the LocusZoom tool (Boughton et al., 2021). Fine mapping was performed using the FUnctional Mapping and Annotation (FUMA) toolbox (Watanabe et al., 2017).

### Molecular Biology Experiments

Expression plasmid containing the wild type C-terminally myc-DDK tagged HAP1 cDNA under the control of the constitutive CMV promoter or the corresponding *Hap1^K4R^* (rs4796604 G > A) variant were transfected in the human neuroblastoma derived cell line, SH-SYS5 (ATCC CRL-2266) cells. Cells and supernatant were harvested 48 h after transfection. For protein analysis, cell lysis supernatant were recovered after 10 min centrifugation at 10,000 × *g*. Sub-cellular fractionation were performed using the Subcellular Protein Fractionation Kit for Cultured Cells (ThermoFisher Scientific). All protein preparations were separated by SDS-PAGE and analyzed by Western-blotting. mRNA expression levels were analyzed by RT-PCR with normalization to ACT1 mRNA. All molecular biology experiments were done in triplicates and similar transfection efficiency controlled using a co-transfected fluorescent construct. Human HAP1 expression data where extracted from the NCBI Gene Expression Omnibus (GEO) and analyzed using the GEO2R tool for comparison (Edgar et al., 2002; Barrett et al., 2013). Detailed protocols for cell culture experiments are available in Supplementary Methods 3.

### Statistical Analysis

Descriptive statistics, group comparisons, multicollinearity variance inflation factor (vif) and linear regressions for sensitivity analyses were done in R version 3.5.3 (Johnston et al., 2018; Fox and Weisberg, 2019). The Mann–Whitney–Wilcoxon (numerical variables) and Chi-square (categorical variables) statistics were used for pairwise comparison. The Fischer’s exact test was used for higher order categorical variables, while correlation between ordinal or numerical variables were tested using the Kendall rank correlation coefficient test. The results of statistical tests with *p*-values ≤ 0.05 were considered significant. The Bonferroni correction for multiple testing was applied where appropriate. Sensitivity analyses were performed by comparing the changes of the estimates (beta values) resulting from calculating the linear regression parameters either in a subgroup of the study population (by removing patients taking comedication, considering only FMS patients or patients above 40 years old) or removing a variable form the analysis (for no diagnosis) (Thabane et al., 2013). The sensitivity analysis was considered successful is the deviation was below 12%.

## Results

### Population Data and Genome-Wide Associations Study Set-up

**FIGURE 1:**
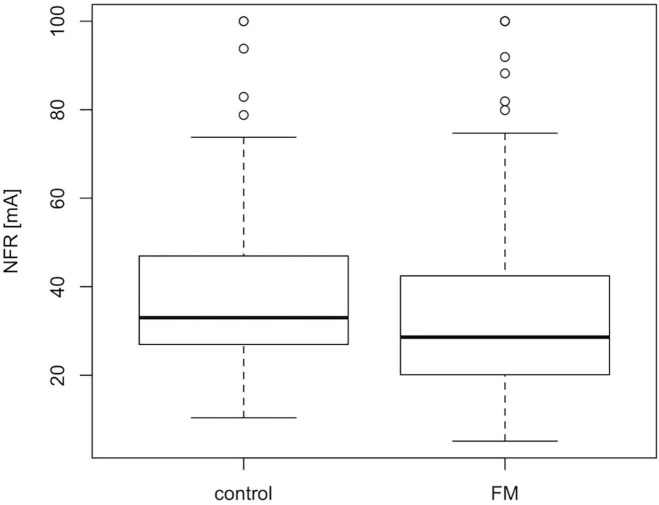
Median NFR threshold is slightly lower in FMS patients than controls. Boxplot showing the median and IQR NFR thresholds values for FMS patients and controls included in the final GWAS population. The difference between the two populations is small but statistically significant according to the Mann–Whitney–Wilcoxon test, *p*-value <0.01; *W* = 6072.

The characteristics of the final study population (212 FMS patients and 72 controls) used for the genomic analysis are described in Table 1. The gender distribution in the patient group is expected as FMS is known to affect primarily women and the proportion of men in the control population is similar to the patient group [5 versus 3%, χ^2^ (1, *N* = 284) = 0.27, *p* = 0.60]. All patients were asked to stop medication susceptible to affect the NFR threshold, and while withdrawal was a condition for further participation in the original clinical studies (Desmeules et al., 2003; Cedraschi et al., 2004; Matthey et al., 2013), all participants with a complete NFR recording and DNA sampling were included in the current genomic study. Drugs affecting NFR threshold in patients unable to stop their medication (USM) were identified according to Sandrini et al. (2005). As expected from previous studies (Desmeules et al., 2003; Tanwar et al., 2019), there is a significant difference (Mann–Whitney–Wilcoxon test, *p* < 0.01; *W* = 6072) in NFR threshold values between the FMS patient and the control groups (Figure 1).

**TABLE 1:** Genome-wide association study population.

	FMS patients	Controls	Total
Individuals	212	72	284
Age (min. − max.)	52 ± 11 (18–81)	50 ± 11 (29–75)	51 ± 11 (18–81)
Cohort of origin (1 = AquaFM, 2 = Milna, 3 = PNR)	1:48 2:96 3:68	1:21 2:0 3:51	1:69 2:96 3:119
Mean NFR ± SD	34.8 ± 22.4	39.9 ± 21.0	36.1 ± 22.1
Median NFR ± IQR*^a^*	28.6 ± 22.2	33.0 ± 19.8	30.2 ± 21.7
Gender	11 males, 201 females	2 males, 70 females	13 males, 271 females
Participants with co-medication	Yes: 69 No: 143	Yes: 2 No: 70	Yes: 71 No: 213

*^a^Mann–Whitney–Wilcoxon test: p-value < 0.01, W = 6072.*

Due to deviation from normality of the NFR threshold values distribution (Supplementary Data 4), log_10_ transformed values were used for regression analysis. The co-variates selected for multiple regression analysis were age, gender, FMS diagnosis, cohort of origin, and presence of co-medication susceptible to affect the NFR threshold. There was no relevant correlation between any of those variables and the 10 first axes of the PCA of the genetic data (Supplementary Data 5). An additive genetic model was assumed.

### Genome-Wide Association Study Results

**FIGURE 2:**
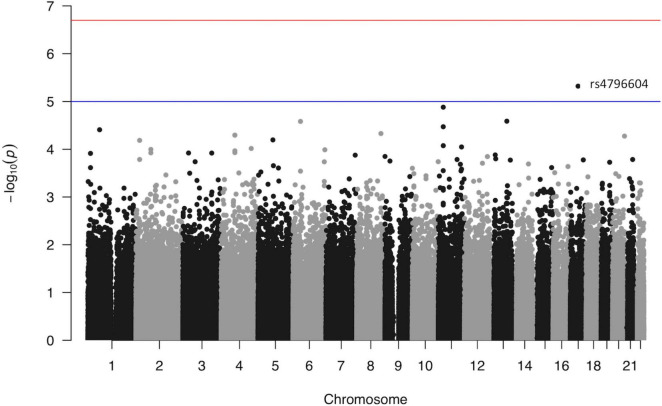
Manhattan plot from the linear regression analysis of the GWAS versus log_10_(NFR thresholds). Each point represents one single nucleotide polymorphism ordered according to its genomic position (*x* axis). The *y* axis reflects the probability value [expressed as −log_10_(value)] obtained from the logistic regression analysis of each individual SNP in the study. The red line marks the genome-wide association *p*-value threshold level (2E−07). The blue line marks an arbitrary level set at 1E−05.

**FIGURE 3:**
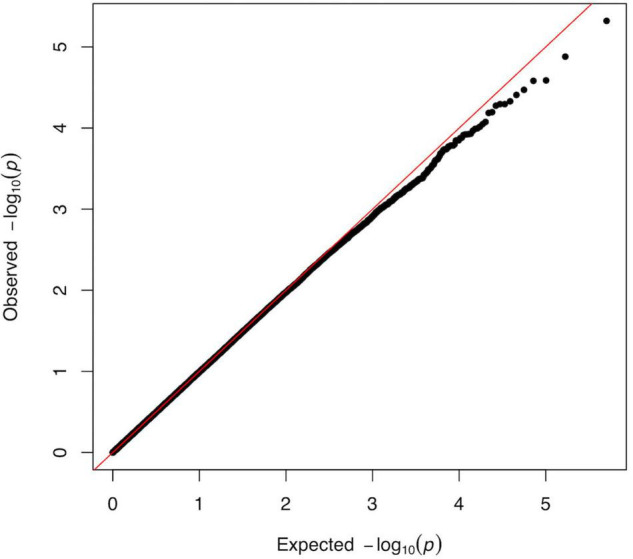
QQ-plot from the linear regression analysis of the GWAS versus log_10_(NFR thresholds). Plot of observed versus expected distribution of *p*-values across all SNPs of the GWAS analysis. The predicted *p*-value is determined as a normally distributed set of probability statistics based on the number of SNPs included in the study (Turner, 2014). The graph shows no major deviation from normality (diagonal) and suggest the absence of confounder in the data. The tendency observed for the lowest *p*-values indicating that we identified less highly specific targets than might have been expected from a completely random distribution, could reflect the high complexity of the trait as well as the genetic redundancy underlying fundamental neuronal processes. The genetic inflation statistics (λ_*GC*_ = 0.989, λ_1000_ = 0.962) reveals no systematic population stratification bias in our analysis.

The association *p*-values for SNPs located on autosomal chromosomes and the NFR threshold are shown in Figure 2. The corresponding QQ-plot displayed in Figure 3 and inflation statistics with λ_*GC*_ = 0.989, shows that there is no notable evidence of population substructure. The top hits from the linear regression analysis are shown in Supplementary Data 6. No SNPs located on the X chromosome appeared in the top 20 results and the Y chromosome was not investigated due to the low proportion of men in the cohort.

None of the candidates from the genome-wide analysis of the cohort reaches the threshold set by the Bonferroni correction (i.e., 0.05/258,756 = 2E−07). However, both the highly polygenic nature of pain signaling, and the low number of samples included in the study could account for the low association statistics. In addition, the Bonferroni correction is often considered too stringent for genome-wide analyses as SNPs are not entirely independent from each other and using the method presented in Li et al. (2012) to evaluate the effective number of tests gives a suggestive *p*-value threshold of 5.95E−06 for the present study. The top hit of our analysis and only SNP to reach this threshold is rs4796604 (A > G) (*p* = 4.78E−06) located on chromosome 17.

**FIGURE 4:**
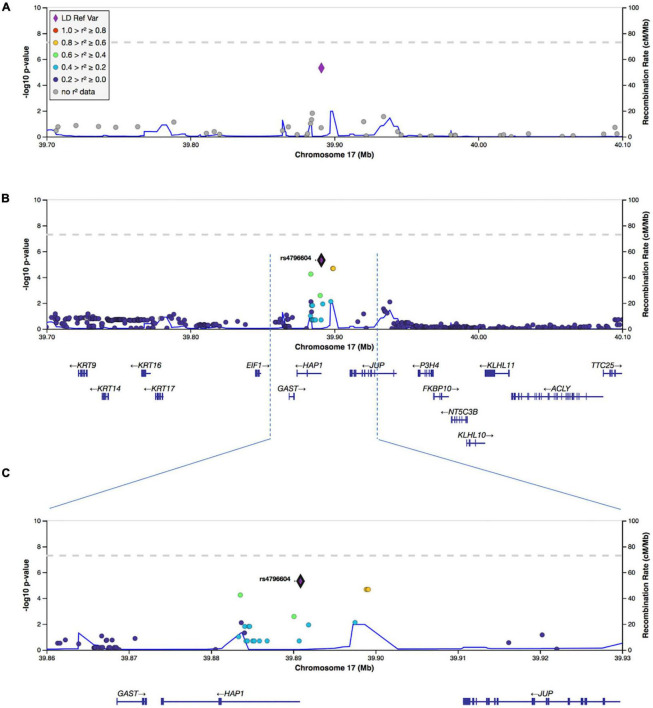
Regional visualization plots of GWAS results surrounding lead SNP rs4796604. **(A)** Association results from the original GWAS analysis. **(B)** Association results following imputation of chromosome 17. **(C)** Zoomed in window (39,860,000–39,930,000) for imputed chr17 results.

To increase the SNP coverage around rs4796604, we performed the imputation on chromosome 17 and obtained a dataset of 348,128 SNP. Fine mapping of the association results using the imputed data, confirmed rs4796604 as the lead SNP of our analysis as shown on the regional association plots displayed in Figure 4.

Different sensitivity analyses were used to test the robustness of the association between rs4796604 and log_10_(NFR). We found the association to be consistent with respect to comedication (excluding patients taking comedications), FMS diagnosis (using either FMS patients group alone or excluding the diagnosis from the linear regression model) and age (excluding participants below 40 years old).

Gene mapping shows that the SNP rs4796604 corresponds to a non-synonymous variant located inside the *HAP1* gene (chr17, MAF of 0.5 in Europeans). HAP1 is mainly expressed in neuronal cells (Li et al., 1995, 1998b; Gutekunst et al., 1998). It has been associated with microtubule-dependent transporters (Twelvetrees et al., 2019), neurotrophic growth factors trafficking and recycling of internalized membrane receptors including TrkA, TrkB, and GABA_*A*_R (Li et al., 2002; Gauthier et al., 2004; Kittler et al., 2004; Rong et al., 2006; Sheng et al., 2006, 2008; Twelvetrees et al., 2010; Wu et al., 2010; Yang et al., 2011; Xiang et al., 2014; Mele et al., 2017; Lim et al., 2018) in humans, as well as catecholamine and glutamate release from mouse chromaffin cells (Mackenzie et al., 2016).

### Log_10_ NFR Thresholds Correlate With rs4796604 Genotype, Fibromyalgia Syndrome Diagnosis, and Presence of Co-medication

We used a multiple linear regression analysis including five co-variates (age, gender, FMS diagnosis, presence of co-medication, and cohort of origin) to assess the GWAS data. The parameters of the model for the association between the rs4796604 genotype and log_10_ transformed NFR threshold levels are displayed in Table 2. The resulting regression model equation is significant (*F*_6,277_ = 8.047, *p* = 5.08E−08) with an adjusted *R*^2^ of 0.130 that is consistent with the complexity of the trait. All variance inflation factors (vif) of the multicollinearity analysis are <1.2, well below accepted thresholds indicating the presence of collinearity in the data.

**TABLE 2:** Multiple linear regression model parameters for the association between log_10_(NFR threshold) and rs4796604.

	Beta	CI (95%)	*Z*-score	*T* stat	*p*-Value
Intercept	1.328	1.115 to 1.542	4.71E−17	0.000	1.00E−00
rs4796604 (AA = 1, AG = 2, GG = 3)	0.091	0.053 to 0.130	0.261	4.666	4.78E−06***
FMS diagnosis (no = 1, yes = 2)	–0.125	−0.196 to −0.054	−0.208	–3.457	6.32E−04***
Gender (women = 0, men = 1)	–0.036	−0.174 to 0.103	−0.028	–0.506	6.13E−01
Age (years)	0.001	−0.002 to 0.004	0.042	0.745	4.57E−01
Co-medication (no = 1, yes = 2)	0.134	0.063 to 0.206	0.222	3.688	2.72E−04***
Cohort (AquaFM = 1, Milna = 2, PNR = 3)	–0.011	−0.049 to 0.026	−0.035	–0.594	5.53E−01

Beta, regression coefficient; CI (95%), lower-upper limit of the 95% confidence interval; Z-score, standardized regression coefficient; T stat, coefficient t-statistics; p-value, asymptotic p-value for t-statistics. ***p < 0.001.

The individual parameters of each variable show that the presence of co-medication and FMS diagnosis are important co-variates of the model while neither age nor gender bring significant contributions. It is noteworthy that the cohort of origin does not introduce any significant bias although NFR measurements were spread from 1998 to 2009.

The parameters of the standardized regression model suggest that the three significant parameters share similar weights, with the presence of any additional G allele at position rs4796604 increasing the NFR value by an factor of 0.261. In comparison, the presence of co-medications increases the threshold with a standardized beta of 0.222, while FMS diagnosis decreases the threshold by an standard estimate of 0.208 versus the control population. The NFR threshold values and distribution for the different groups in our study according to rs4796604 genotype, FMS diagnosis and presence of co-medication are presented in Table 3.

**TABLE 3:** NFR threshold distribution according to significant study parameters.

	rs4796604 genotype	All		ASM		USM	
		
		*N*	NFR	*N*	NFR	*N*	NFR
Full cohort	Total	284	30.2 (±21.7)	213	28.4 (±20.0)	71	38.8 (±24.2)
	AA	82	23.1 (±17.1)	67	23.0 (±15.3)	15	33.6 (±22.3)
	AG	124	33.3 (±18.6)	91	29.9 (±23.1)	33	38.8 (±23.0)
	GG	78	35.6 (±33.3)	55	30.2 (±31.3)	23	48.1 (±39.2)
*p*-value			6.12E−07		1.07E−04		7.50E−03
FMS	Total	212	28.6 (±22.2)	143	25.1 (±17.4)	69	38.8 (±23.9)
	AA	60	21.1 (±14.5)	45	19.1 (±12.5)	15	33.6 (±22.3)
	AG	98	33.1 (±17.2)	65	28.5 (±15.2)	33	38.8 (±23.0)
	GG	54	34.4 (±34.1)	33	25.7 (±28.1)	21	48.1 (±39.5)
*p*-value			3.01E−06		6.92E−04		5.16E−03
Controls	Total	72	33.0 (±19.8)	70	33.0 (±19.4)	2	36.8 (±14.6)
	AA	22	30.9 (±10.0)	22	30.9 (±10.0)	–	na
	AG	26	34.8 (±21.9)	26	34.8 (±21.9)	–	na
	GG	24	40.4 (±37.6)	22	40.4 (±38.6)	2	36.8 (±14.6)
*p*-value			7.18E−02		6.03E−02		na

*NFR: median values (±IQR). N, number of participants; FMS, fibromyalgia patients; ASM, able to stop medication; USM, unable to stop medication; p-values for Kendall rank correlation coefficient test.*

### Relationship Between Nociceptive Flexion Reflex Threshold, Fibromyalgia Syndrome Diagnosis, and rs4796604 Genotype

**FIGURE 5:**
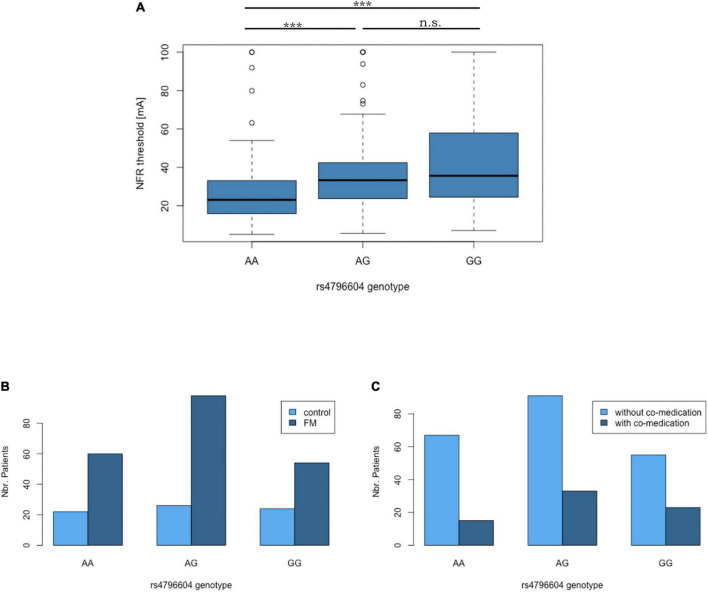
The rs4796604 A allele correlates with lower NFR threshold values. **(A)** Median NFR threshold distribution in function of rs4796604 genotypes. Mann–Whitney statistics were used to assess the differences between genotypes (with ***p < 0.001). There is no correlation between rs4796604 genotype and FMS diagnosis **(B)**, nor presence of co-medication **(C)**. n.s. = not significant.

The correlation between the rs4796604 genotype and NFR thresholds is highly significant (*r*_*T*_ = 0.23, *p* = 6.12E−07, Kendall’s Tau statistics). Moreover, according to the Mann–Whitney *U* test, the rs4796604 AA genotype group has significantly lower NFR threshold values than the AG and GG groups (Figure 5A).

In addition, although the correlation between the rs4796604 genotype and the NFR threshold is visible in every subgroup, there are no significant differences between the subgroups (i.e., FMS versus controls or with and without co-medication) based on rs4796604 genotype except, in the most severely affected AA group, where FMS patients have significantly lower NFR thresholds than the controls (*p* < 0.01) (Supplementary Data 7).

Thus, our genome-wide analysis identified the SNP rs4796604 as a putative candidate for the prediction of NFR threshold levels. However, there is no correlation between this polymorphism and FMS diagnosis or the presence of co-medication using the Fisher exact test (Figures 5B,C). We found no correlation between rs4796604 genotype and any other clinical scale available from the original studies records (Desmeules et al., 2003; Cedraschi et al., 2004; Matthey et al., 2013), such as pain or FMS severity as evaluated through quantitative sensory testing (QST) (hot and cold temperature pain thresholds, cold pressor test), or questionnaires assessing the impact of FMS on the patient’s quality-of-life (Fibromyalgia Impact Questionnaire – FIQ), Psychological General Well-Being (PGWB), or functional status (Physical Component Score of the SF-36). However, as previously reported in the literature (Neziri et al., 2011), although all mentioned QST results, except cold and warm perception thresholds, showed a highly significant difference between case and controls, they are only poorly correlated with NFR threshold values. The summary of the QST results in our cohort and their correlation with NFR values is available in Supplementary Material 8.

### Effect of the rs4796604 Mutation on Huntingtin Associated Protein 1 (HAP1) Transcription, Protein Expression, and Sub-cellular Distribution

The rs4796604 A > G polymorphism is located within the first exon of the *HAP1* gene and results in a lysine (K) to arginine (R) amino-acid substitution in position 4 of the encoded protein. The SNP rs4796604 is located at the very N-terminus of the protein and in close proximity to a N-myristylation site. Myristoyl is a lipid tail used for membrane or protein interaction which is often involved in sub-cellular localization of the attached protein (Martin et al., 2011). Thus, we decided to investigated the difference in protein levels and the cellular distribution of the mutant versus the wild-type protein. We therefore transfected the neuroblastoma-derived SH-SYS5 cells with plasmids constitutively overexpressing a C-terminal DDK-myc tagged version of the Hap1*^K4R^* mutant and corresponding wild-type protein. Cells were harvested 48 h post-transfection and both the mRNA and protein content were analyzed.

RT-PCR analysis of mRNA extracts showed that the expression levels of both constructs are similar in the chosen expression system. In parallel, we compared *HAP1* mRNA expression levels in function of the rs4796604 genotype in a human reference population of European descent (CEU) using global expression dataset available from the GEO public repository [(Huang et al., 2007), accession number: GSE7761] and the GEO2R tool. There were no differences in *HAP1* mRNA expression based on rs4796604 polymorphism among the 82 participants included (22 AA, 44 AG, and 16 GG with a MAF of 0.54, *p* = 0.33).

Total protein expression and subcellular distribution were assessed using the C-terminal Flag-tag of the constructs. As shown in Supplementary Data 9, and in line with the expression data, the Hap1*^K4R^* mutation did not affect the overall protein amount when compared to actin expression levels. As expected from previous studies, the major portion of the wild-type protein is found associated with secretory vesicles and/or cytoskeletal fraction (Li et al., 1996, 1998a; Engelender et al., 1997; Gutekunst et al., 1998). The Hap1*^K4R^* mutation has no visible effect on subcellular protein distribution under the current experimental conditions (Supplementary Data 9).

## Discussion

Our GWAS identified rs4796604, a polymorphism resulting in an amino-acid substitution in the *HAP1* gene, as a potential new genetic determinant of the NFR threshold, a measure of enhanced central pain signaling. The linear regression model parameter obtained for rs4796604 shows that FMS diagnosis and the presence of concomitant medication are important co-factors, while neither age nor gender have a significant effect. The influence of FMS diagnosis is in line with previous studies reporting a lowering of the threshold in FMS patients compared to a control population (Desmeules et al., 2003; Banic et al., 2004; Tanwar et al., 2019). Similarly, many pharmacological compounds affecting neurotransmission in the CNS are known to have a strong influence on NFR thresholds (Skljarevski and Ramadan, 2002; Sandrini et al., 2005). Although gender influence on NFR thresholds has been previously reported, the low proportion of men in our cohort might mask this effect.

Central sensitization results from an acquired imbalance in pain-transmitting and pain-attenuating signals in the CNS. Many different neuro-transmitters (NT) such as catecholamines, glutamate and GABA are involved in transmission and down-regulation of nociceptive stimuli in nerve terminals (reviewed by Harte et al., 2018). Moreover, neuronal growth factors as BDNF or nerve growth factor (NGF) play an important role not only in the development but also in the maintenance of different neuronal cell populations that are important for the regulation of nociceptive stimuli (Dai and Zhou, 2014). Although the precise molecular function of Hap1 in neuronal cells remains unclear, this microtubule-associated protein is involved in intracellular trafficking in neurons and participates both in neurotransmitter (NT) release (Mackenzie et al., 2016) and plasma membrane receptor recycling *via* endocytic compartments (Li et al., 2002; Kittler et al., 2004; Rong et al., 2006; Sheng et al., 2008; Twelvetrees et al., 2010; Xiang et al., 2014) possibly by acting as a scaffold protein (Rong et al., 2007). HAP1 expression is particularly enriched in the hypothalamus, although *HAP1* mRNA is detected in all brain tissues and the spinal cord.^2^

HAP1 was first identified as an interactor of neurodegenerative Huntingtin (Htt), the protein responsible for Huntington disease and was subsequently shown to protect specific neuronal cells from degradation (Li et al., 2003; Mele et al., 2017; Wroblewski et al., 2018). Moreover, HAP1 has many connections to nociceptive signal transduction. First, HAP1 plays a role in the NGF-receptor TrkA recycling to the plasma membrane (Rong et al., 2006). NGF promotes neurite outgrowth in developing cells and is required for survival of sensory neurons (Marmigere et al., 2006). Interestingly, intramuscular injections of NGF are able to induce neuronal sensitization in rats (Hoheisel et al., 2007) and some mutations in TrkA result in congenital insensitivity to pain with anhidrosis (CIPA), an autosomal recessive disorder characterized by the inability to feel pain and absence of sweating (Indo et al., 1996). HAP1 also participates in regulating neuronal excitability through the recycling of GABA_*A*_ receptors to postsynaptic membranes (Kittler et al., 2004; Sheng et al., 2006; Twelvetrees et al., 2010; Mele et al., 2017). Inhibitory mechanisms regulated by GABA and GABA-receptors in the spinal dorsal horn play a central role in modulating pain signals transmitted to the brain (Prescott, 2015), and the strength of the inhibitory signal at synapses correlates with the amount of GABA_*A*_ receptors present on the membrane (Jacob et al., 2008). Pharmacological modulation of GABA_*A*_ receptor mediated signaling is considered an interesting alternative for pain management in chronic pain conditions. However, the sedative properties of currently available GABA_*A*_ receptor agonists limit their use as analgesics (Fischer, 2017). In a slightly different register, HAP1 was also shown to participate directly in exocytosis and regulate plasma membrane docking of secretory vesicle from mouse neuroendocrine cells thereby regulating release of catecholamines and glutamate from synapses (Mackenzie et al., 2016). While glutamate is the main excitatory NT in human CNS acting through NDMA-receptors to transmit peripheral nociceptive signals to the brain, catecholamines, as serotonin or norepinephrine, are the mediators of descending pain modulatory signals transmission playing an important role in pain attenuation (Pereira and Goudet, 2018; Bravo et al., 2019). Finally, Hap1 is involved both in proBDNF anterograde transport, and endocytosis of the BDNF-TrkB receptor complex (Gauthier et al., 2004; Sheng et al., 2008; Wu et al., 2010; Yang et al., 2011; Xiang et al., 2014; Lim et al., 2018). BDNF is a neurotrophic growth factor promoting neuronal growth and survival, as well as synaptic plasticity influencing memory, learning and cognition processes (Sasi et al., 2017). BDNF acts as an interesting modulator of nociception and has been involved in central sensitization, although its pleiotropic roles at different levels of the CNS are hard to interpret (Merighi et al., 2008; Sasi et al., 2017). Indeed, internalization of the BDNF-TrkB complex triggers a variety of signaling cascades modulating both excitatory (glutamine) and inhibitory (GABA) signaling pathways (Sasi et al., 2017). Thus, many nociceptive signal transduction pathways are connected to HAP1 function. In addition, in a completely different approach, the authors of a recent immunolabeling study showed an important overlap between HAP1 and different neurochemical markers in the mouse dorsal root ganglion (DRG) neurons and suggested a role for HAP1 in pain transduction (Islam et al., 2020).

Although Hap1 rs4796604 genotype correlates with levels of the NFR threshold, we found no direct link between Hap1 and FMS diagnosis according to ACR 1990 criteria. However, many of the processes regulated by Hap1 have been linked with FMS and are believed to participate in the onset or the progression of the condition, suggesting that understanding Hap1 cellular activity could help specify the nature of the central sensitization process in different FMS patients. Indeed, reflecting the broad range of functions mediated by neurotrophic growth factors like BDNF and NGF, their implication in FMS has been postulated at many different levels, from regulation of neurotransmission to synaptic plasticity affecting learning and memory processes. However, while the involvement in pain signaling of those factors is well documented, if not well understood, their implication in FMS is less clear. Indeed, differences in BDNF and NGF circulating and cerebrospinal fluid (CSF) levels between FMS patients and healthy controls are still controversial although most studies report increased concentrations (Baumeister et al., 2019; Brietzke et al., 2019). However, the expression pattern of neurotrophic factors are not uniform across different regions of the CNS (Notaras and van den Buuse, 2020), thus the physiological implications of increased peripheral or even central BDNF or NGF levels remain unclear. On the genetic level, a study associated different BDNF related polymorphisms with FMS in the Korean population (Park et al., 2018). A recent study showed increased GABA_*A*_R levels in cortical neurons of FMS patients (Pomares et al., 2020). Regarding NTs in FMS, both serotonin and norepinephrine levels were found to be significantly decreased in CSF of FMS patients (Russell et al., 1992), while glutamate levels are increased in specific brain areas of FMS patients (Foerster et al., 2015). In addition, the most common pharmacological treatments used in the context of FMS are inhibitors of the NMDA glutamate receptor or of serotonin and/or norepinephrine reuptake (Sluka and Clauw, 2016; Pereira and Goudet, 2018; Bravo et al., 2019).

Despite the observed heritability of pain-related diseases, the search for polymorphisms associated with chronic pain conditions in general and FMS in particular is a long-standing issue. Candidate gene approaches focused their attention on SNPs affecting neurotransmitters activity known to be relevant for pain signal transduction and processing as well as on endogenous pain modulating systems (reviewed by Knezevic et al., 2018; Janssen et al., 2021). Frequently reported genes include serotonin receptors and transporter (*HTR*s, *SLC6A4*), dopamine receptor and degradation (*COMT*, *TAAR1*), glutamate receptor (*GRIA4*), adrenergic receptors (*ADR*s) as well as the mu opioid receptor *OPRM1*, the NGF *BDNF* or sodium channels (*TRVP*s for instance). In addition to candidate approaches, several genome-wide association studies directed toward elucidation of the genetic background of FMS, chronic widespread pain (CWP) or related chronic pain conditions uncovered additional candidate genes and polymorphisms (Docampo et al., 2014; Johnston et al., 2019; Rahman et al., 2021). None of the polymorphism previously associated with FMS or other chronic pain conditions nor any of the more recent GWAS candidates did show significant association with NFR in our study (data not shown). This is not unexpected as the NFR threshold, while being intimately linked to pain signal transmission is not a proxy for FMS nor CWP.

Pain perception is a highly polygenic trait that is very difficult to evaluate accurately, and although measurement of NFR thresholds constitutes one of the most objective assessment of the central pain signaling network reactivity available, a number of modulating factors ranging from pharmacological interactions to mental and psychological states have been identified (Sandrini et al., 2005). Thus, taking all those considerations into account, the number of participants in the current study is low and could easily account for the poor statistical power of our GWAS results, which is clearly the major limitation of the current study. Additional limitations to the current study, include the recognized inter- and even intra-individual variability of NFR measurements that are affected by a number of environmental and psychological factors in addition to the physical parameters as well as the low proportion of men in our cohort or the use of the old ACR 1990 criteria for FMS diagnosis.

The replication of our findings in independent cohorts will be critical to confirm their validity. Considering the measured effect size, a MAF of 0.5 in a European population and a significance threshold of *p* = 0.05, the replication of rs4796604 association with NFR would require a sample size of about 200 participants (power calculations heatmaps according to Visscher et al. (2017) are presented in Supplementary Data 10). Moreover, characterization of the functional implication of the *Hap1^Lys4Arg^* mutation at the molecular level will be central to unravel the importance of this variant for transmission of painful stimuli and understand whether there is a physiological relevant difference in nociceptive signal transmission between individuals expressing different forms of the protein. Although the position of the mutation at the very beginning of a N-myristoylated protein could potentially interfere with Hap1’s membrane attachment, we could not observe any differences in expression nor subcellular repartition between the wild type and mutant protein under current experimental conditions. However, N-myristoylation is often associated with dynamic membrane attachment which might not be apparent in a steady-state distribution. Alternatively, the high expression levels resulting from constitutive expression of the exogenous transcript could mask or disrupt the normal cellular pattern.

In summary, using a GWAS, we identified a new potential genetic determinant of NFR threshold level. While Hap1 is not a predictor of FMS, this protein is involved in the regulation of several signaling pathways involved in the pathophysiology of the disease, and differential regulation of synaptic transmission through modulation of intracellular vesicular transport at synapses might help to better characterize the enhanced sensitization in individual patients. Further studies will be directed at replicating the current results in independent cohorts, as well as unraveling of the functional implications of the *Hap1^K4R^* mutation in Hap1 protein function.

## Data Availability Statement

The datasets presented in this study can be found in online repositories. The names of the repository/repositories and accession number(s) can be found below: https://doi.org/10.26037/yareta:l35qwdy5t5b5vll3ae3hmzigy4.

## Ethics Statement

The studies involving human participants were reviewed and approved by the Geneva Ethics Committee – CCER (Commission Cantonale d’Ethique de la Recherche) de la République et du Canton de Genève, Geneva, Switzerland. All patients/participants provided their written informed consent to participate in this study.

## Author Contributions

YG, JD, AM, and EP designed the study. YG, KS, and MM performed the experiments. YG, KS, and GE performed the bioinformatic analysis. YG wrote the manuscripts’ draft and prepared the figures and tables. JD, MM, EK, GP, EP, CC, and GE reviewed the draft. All authors read and approved the final manuscript.

## Conflict of Interest

The authors declare that the research was conducted in the absence of any commercial or financial relationships that could be construed as a potential conflict of interest.

## Publisher’s Note

All claims expressed in this article are solely those of the authors and do not necessarily represent those of their affiliated organizations, or those of the publisher, the editors and the reviewers. Any product that may be evaluated in this article, or claim that may be made by its manufacturer, is not guaranteed or endorsed by the publisher.

## Footnotes

1 Mouterde, M., Daali, Y., Rollason, V., Čížková, M., Mulugeta, A., Al-Balushi, K. K., et al. (under review) Joint analysis of phenotypic and genomic diversity sheds light on the evolution of xenobiotic metabolism in humans. *Genome Biology and Evolution*.

2
https://gtexportal.org
