# Supplementary material for: Uncovering a Genetic Polymorphism Located in Huntingtin Associated Protein 1 in Modulation of Central Pain Sensitization Signaling Pathways
Source: Front Neurosci. 2022 Jun 28;16:807773. doi: 10.3389/fnins.2022.807773 (PMC9274135; doi:10.3389/fnins.2022.807773)
Supplement: Supplementary file 2 [file Data_Sheet_2.DOCX]

**Supplementary Data S2: Genotyping results quality control**

|  | Nr SNPs |
| --- | --- |
| Final data set | 258’756 |
| Geno > 0.02 | 11’112 |
| HWE p-value < 10e-5 | 79 |
| MAF < 0.05 | 289'904 |
| Total | 555’356 |

**Relatedness check for clean data set** with IBD (identity by descend)

All samples with PI_HAT > 0.15

| ID_1 | ID_2 | Z0 | Z1 | Z2 | PI_HAT |
| --- | --- | --- | --- | --- | --- |
| BM00181 | *BM00227* | 0.4629 | 0.5327 | 0.0043 | 0.2707 |
| BM00181 | BM00337 | 0.5027 | 0.4832 | 0.014 | 0.2556 |
| *BM00225* | BM00328 | 0.2762 | 0.4867 | 0.2371 | 0.4804 |
| *BM00227* | BM00337 | 0.2307 | 0.5346 | 0.2347 | 0.502 |
| *BM00048* | BM00049 | 0 | 1 | 0 | 0.5 |

| ID | Call rate |
| --- | --- |
| BM00337 | 0.9848 |
| *BM00227* | 0.9847 |
| *BM00225* | 0.9805 |
| BM00328 | 0.9808 |
| *BM00048* | 0.9846 |
| BM00049 | 0.9847 |

Note: for completely unrelated people z0 =1, for identical twins z2=1

Half of the samples with 1^st^ degree family relation (ie 3 volunteers) were excluded based on call rate (in light gray italic in the list)

**Sex check**

X chromosome inbreeding estimate >.95 for males and between -0.4 and +0.4 for females

**Heterozygosity and inbreeding coefficient for autosomal genes**

Heterozygosity rates are expected between 0.35 and 0.45.

**Genotype consistency and duplicate repeatability**

Illumina Chip sequencing contained 6 duplicated samples (2/plate) -> IBD analysis on full GWAS set including + 6 duplicates (named a/b)

| Duplicate ID | "Z0" | "Z1" | "Z2" | PI_HAT | Nbr true missmatch | Total nbr of SNPs genotyped | Percent missmatch |
| --- | --- | --- | --- | --- | --- | --- | --- |
| "Duplicate 1" | "0.0000" | "0.0000" | "1.0000" | "1.0000" | 4 | 551364 | 7.25E-04 |
| "Duplicate 2" | "0.0000" | "0.0000" | "1.0000" | "1.0000" | 2 | 551439 | 3.63E-04 |
| "Duplicate 3" | "0.0000" | "0.0000" | "1.0000" | "1.0000" | 2 | 551287 | 3.63E-04 |
| "Duplicate 4" | "0.0000" | "0.0000" | "1.0000" | "1.0000" | 0 | 551310 | 0.00E+00 |
| "Duplicate 5" | "0.0000" | "0.0000" | "1.0000" | "1.0000" | 3 | 551302 | 5.44E-04 |
| "Duplicate 6" | "0.0001" | "0.0000" | "0.9999" | "0.9999" | 4 | 551205 | 7.26E-04 |

NB if unrelated people z0 =1, if identical twins z2=1

**Allele frequency consistency with HRC EUR population**

with the European population from the Haplotype Reference Consortium^1^ (HRC version r1.1, 2016, available from the Michigan Imputation Server^2^)


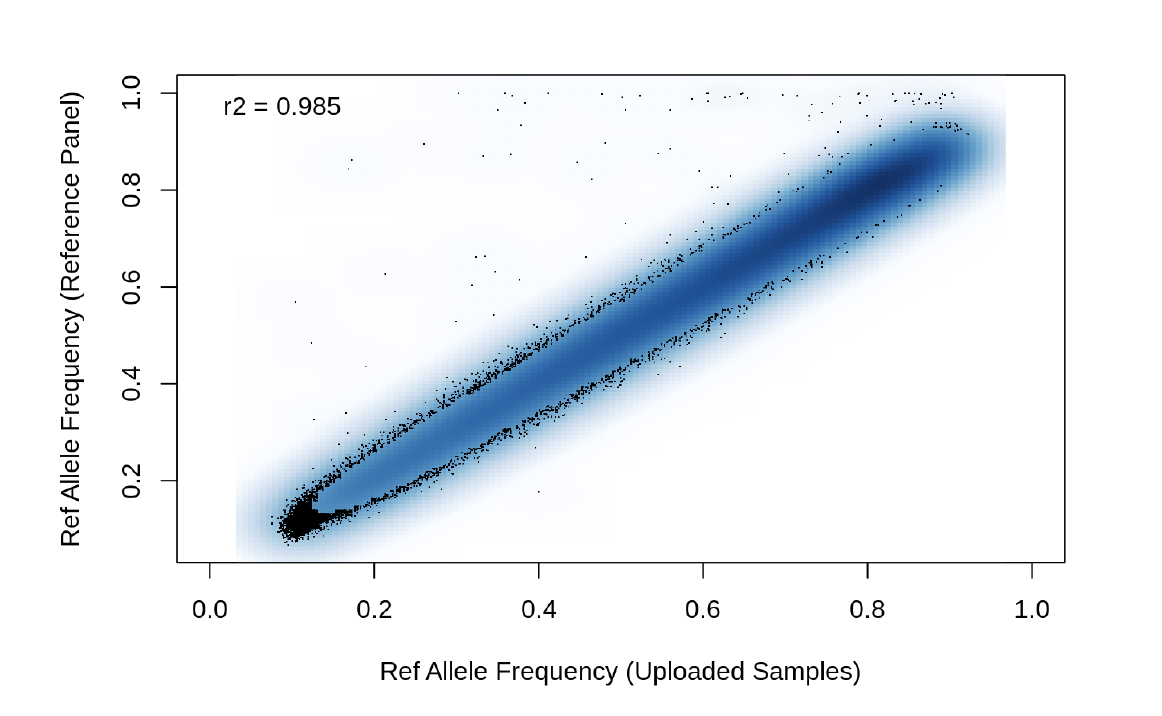


Linear regression analysis: (V1 =HRC-EUR, V2=GWAS-FM). 🡪R^2^ = 0.985

**Ethnicity check –** PCA with HapMap data Phase III^3-5^ release 2, available from NCBI

Cut off limit

| PCA axis | 1 | 2 |
| --- | --- | --- |
| Weight [%] | 52.00 | 26.16 |

Samples with > 30% deviation from EUR population on the primary and secondary axis (PC1 and PC2) of the PCA analysis (ie 15 volunteers) were excluded.

References:

^1^ http://www.haplotype-reference-consortium.org/home

^2^ https://imputationserver.sph.umich.edu/index.html#!pages/home

^3^ The International HapMap Consortium. A haplotype map of the human genome. Nature. 2005;437: 1299–320. doi:10.1038/nature04226

^4^ The International HapMap Consortium. A second generation human haplotype map of over 3.1 million SNPs. Nature. 2007;449: 851. doi:10.1038/nature06258

^5^ The International HapMap Consortium. Integrating common and rare genetic variation in diverse human populations. Nature. 2010;467. doi:10.1038/nature09298
