# Supplementary material for: Uncovering a Genetic Polymorphism Located in Huntingtin Associated Protein 1 in Modulation of Central Pain Sensitization Signaling Pathways
Source: Front Neurosci. 2022 Jun 28;16:807773. doi: 10.3389/fnins.2022.807773 (PMC9274135; doi:10.3389/fnins.2022.807773)
Supplement: Supplementary file 3 [file Data_Sheet_3.docx]

**Supplementary Methods S3: Detailed material and method description for Molecular biology experiments**

*HAP1 expression constructs, cell transfection and harvest*

Expression plasmid containing the wild type C-terminally myc-DDK tagged HAP1 cDNA under the control of the constitutive CMV promoter was purchased from OriGene Technologies Inc. (RC220251). The *Hap1^K4R^* (rs4796604 G>A) point mutation was introduced by PCR directed mutagenesis (see table below for primer sequences) and subcloning into the OriGene Technologies Inc. pENTRY plasmid (PS100001) using restriction enzymes SgfI and XhoI. The presence of the mutation was verified by Sanger sequencing.

The constructs were introduced in the human neuroblastoma derived cell line, SH-SYS5 (ATCC CRL-2266) cells, using the jetPRIME® transfection reagent (Polyplus-transfection® SA). All molecular biology experiments were done in triplicates.

SH-SYS5 cells were grown at 37°C with 5% CO_2_ in DMEM/F-12 + GlutaMAX™ (Dulbecco’s Modified Eagle Medium/Nutrient mixture F-12, ThermoFisher Scientific) media supplemented with 15% decomplemented Fetal Bovin Serum (FBS 10500, ThermoFisher Scientific), penicillin and streptomycin antibiotics (P0781, Merck), 2 mM Glutamine (ThermoFisher Scientific) and 1 % MEM Non-Essential Amino Acid solution (ThermoFisher Scientific). The *HAP1* containing plasmids and the empty vector control were transfected according to manufacturer’s instructions. After 6 hours incubation with the transfection mix, the supernatant was replaced by a maintenance media, identical to the culture media except for the FBS concentration, at 7.5% instead of 15%. Cells and supernatant were harvested 48h after transfection using an 0.05% Trypsin-EDTA solution (Sigma Life Science). Cells were washed in ice cold PBS (spin at 400g, 5 min), aliquoted and pellets processed immediately or frozen at -20°C for protein extraction and sub-cellular fractionation or -80°C for mRNA preparations.

*Total protein extraction, subcellular fractionation and western blot*

For whole protein extraction, frozen cell pellets were resuspended in ice cold lysis buffer (50mM Tris HCl pH 7.8, 10mM NaCl, 0.6% NP-40) containing a protease inhibitor cocktail (cOmplete Mini, EASYpack from Roche) and vortexed for 15 minutes at 4°C. Lysed cells were centrifuged for 10 minutes at 10’000g, 4°C. The supernatant was recovered and boiled for 5 minutes in 1x SDS-PAGE loading buffer.

Sub-cellular fractionations were performed using the Subcellular Protein Fractionation Kit for Cultured Cells from ThermoFisher Scientific (#78840), following manufacturer’s instructions. The resulting fractions were prepared as above for detection.

All protein preparations were analyzed by SDS-PAGE, transferred to nitrocellulose membranes and incubated with respective antibodies. HRP signals were detected using a LAS-4000 imaging system (Fujifilm Life Science, USA). Antibodies used were anti-FLAG (F3165, Sigma-Aldrich), HRP-conjugated anti-actin (ab49900, ABCAM), HRP-conjugated anti-mouse (170-6516, Biorad), anti-Hsp90 (MABC1106, EMD Millipore), anti-pan-PMCA (MABN1802, EMD Millipore) and anti-vimentin (14-9897-80, Invitrogen).

*mRNA extraction and RT-PCR*

Messenger RNA was isolated from frozen cell pellets using the PureLink™ RNA Mini kit (Ambion®), quantified with the Qubit™ RNA BR Assay kit (Invitrogen) and translated to cDNA with the iScript Select cDNA Synthesis kit (Bio-Rad). RT-PCR analysis were performed on a StepOne RT-PCR System (Applied Biosystems) using PowerUp™ SYBR™ Green Master Mix (Applied Biosystems). Primers for the RT-PCR are described in the table below. Normalization was done against ACT1 mRNA expression.

Oligonucleotide Primers Table:

|  | Sequence |
| --- | --- |
| Hap1K4R mutation : |  |
| Forward | 5’ - GAC GCG ATC GCC ATG CGC CCG AGG AGG TT – 3’ |
| Reverse | 5’ – CTG AGA TGA GTT TCT GCT CGA GCG GCC GC – 3’ |
| qPCR : |  |
| ACT1-F | 5’ – Tgg cac cca gca caa tga a – 3’ |
| ACT1-R | 5’ – Cta agt cat agt ccg cct aga agc a - 3’ |
| HAP1-tag-F | 5’ – GCA AGA TGC CCA TTA CAG GC – 3’ |
| HAP1-tag-R | 5’ – TCT TCT GAG ATG AGT TTC TGC TC – 3’ |
