# Supplementary material for: Uncovering a Genetic Polymorphism Located in Huntingtin Associated Protein 1 in Modulation of Central Pain Sensitization Signaling Pathways
Source: Front Neurosci. 2022 Jun 28;16:807773. doi: 10.3389/fnins.2022.807773 (PMC9274135; doi:10.3389/fnins.2022.807773)
Supplement: Supplementary file 4 [file Data_Sheet_4.DOCX]

**Additional file 4: NFR distribution within study population**

|  | NFR | Log_10_ (NFR) |
| --- | --- | --- |
| Mean | 36.10 | 1.48 |
| Stdev | 22.12 | 0.26 |
| Median | 30.15 | 1.48 |
| IQR | 21.65 | 0.30 |
| Min-Max | 5.1-100 | 0.71-2 |
| Skewness | 1.395 | -0.227 |
| Kurtosis | 1.633 | 0.298 |


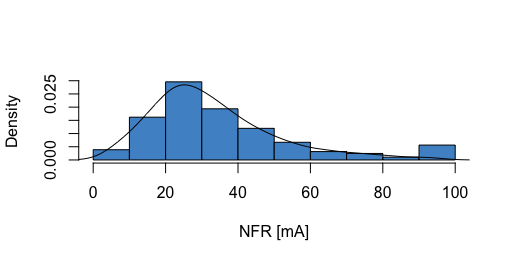

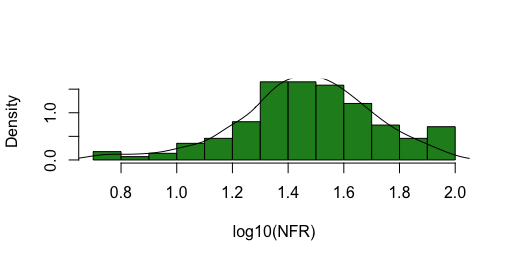


NFR

Note: NFR measurements were limited to 100 mA to preserve participants’ well-being, creating an artificial peak at 100 mA, that was not included in the graphical curve modeling, but is accounted for in the descriptive statistics shown in the table.
