# Supplementary material for: Uncovering a Genetic Polymorphism Located in Huntingtin Associated Protein 1 in Modulation of Central Pain Sensitization Signaling Pathways
Source: Front Neurosci. 2022 Jun 28;16:807773. doi: 10.3389/fnins.2022.807773 (PMC9274135; doi:10.3389/fnins.2022.807773)
Supplement: Supplementary file 5 [file Data_Sheet_5.DOCX]

**Supplementary Data S5: Correlation between genotyping data PCA axes and co-variates**

Calculations were performed in R with the Adegenet package^1,2^ for the PCA analysis of the overall genotyping data.

**Axes weight (from eigenvalues):**

| **x** | **EIG** | **%*** |
| --- | --- | --- |
| 1 | 130.99 | 0.53 |
| 2 | 111.53 | 0.46 |
| 3 | 108.66 | 0.44 |
| 4 | 105.79 | 0.43 |
| 5 | 104.23 | 0.43 |
| 6 | 102.45 | 0.42 |
| 7 | 101.86 | 0.42 |
| 8 | 101.47 | 0.41 |
| 9 | 101.00 | 0.41 |
| 10 | 100.59 | 0.41 |

* percent variation explained by axis

With a significance threshold of 0.005 (5% divided by 10 PCA axes tested) -> only the first axis is significant

**Spearman correlation analysis**

For PCA 1->10 versus

- case/control
- NFR
- cohort of origin (1/2/3)
- age
- gender
- Presence of co-medication (Yes/No)

Sprearman correlation data with correlation coefficient (Rho) and significance (2-tailed sigma value)

|  | FMS/Controls | | NFR threshold  [mA] | | Gender | | Age | | Comedication | | | Cohort of origin | | |
| --- | --- | --- | --- | --- | --- | --- | --- | --- | --- | --- | --- | --- | --- | --- |
|  | Rho | Sigma | Rho | Sigma | Rho | Sigma | Rho | Sigma | Rho | Sigma | Rho | | Sigma |  |
| PC1 | 0.058 | 0.333 | -0.041 | 0.493 | 0.014 | 0.816 | **-0.167** | **0.005** | -0.044 | 0.456 | -0.044 | | 0.456 |  |
| PC2 | -0.018 | 0.763 | -0.013 | 0.823 | -0.104 | 0.079 | 0.009 | 0.874 | -0.042 | 0.481 | -0.042 | | 0.481 |  |
| PC3 | -0.056 | 0.346 | 0.023 | 0.704 | 0.066 | 0.270 | 0.056 | 0.351 | 0.064 | 0.279 | 0.064 | | 0.279 |  |
| PC4 | 0.009 | 0.874 | -0.030 | 0.609 | 0.011 | 0.848 | 0.087 | 0.145 | -0.009 | 0.882 | -0.009 | | 0.882 |  |
| PC5 | 0.059 | 0.323 | -0.017 | 0.779 | -0.028 | 0.633 | -0.010 | 0.865 | -0.041 | 0.495 | -0.041 | | 0.495 |  |
| PC6 | -0.021 | 0.725 | -0.017 | 0.770 | 0.068 | 0.257 | -0.080 | 0.177 | -0.016 | 0.788 | -0.016 | | 0.788 |  |
| PC7 | 0.020 | 0.738 | 0.083 | 0.161 | -0.015 | 0.803 | -0.046 | 0.442 | -0.004 | 0.952 | -0.025 | | 0.952 |  |
| PC8 | -0.054 | 0.367 | 0.078 | 0.189 | -0.080 | 0.180 | 0.063 | 0.287 | 0.098 | 0.099 | -0.023 | | 0.705 |  |
| PC9 | 0.005 | 0.938 | -0.072 | 0.227 | 0.006 | 0.922 | -0.052 | 0.383 | 0.054 | 0.367 | -0.015 | | 0.803 |  |
| PC10 | 0.123 | 0.039 | -0.001 | 0.984 | 0.091 | 0.125 | -0.048 | 0.425 | **0.147** | **0.013** | -0.036 | | 0.541 |  |

with PCx = Primary component axis x

The most significant correlation has a p-value of 0.005 although with a very weak correlation coefficient of -0.167.

References

^1^ Jombart, T. (2008) adegenet: a R package for the multivariate analysis of genetic markers. Bioinformatics 24: 1403-1405. doi: 10.1093/bioinformatics/btn129

^2^ Jombart T. and Ahmed I. (2011) Adegenet 1.3-1: new tools for the analysis of genome-wide SNP data. Bioinformatics. doi: 10.1093/bioinformatics/btr521
