# Supplementary material for: Uncovering a Genetic Polymorphism Located in Huntingtin Associated Protein 1 in Modulation of Central Pain Sensitization Signaling Pathways
Source: Front Neurosci. 2022 Jun 28;16:807773. doi: 10.3389/fnins.2022.807773 (PMC9274135; doi:10.3389/fnins.2022.807773)
Supplement: Supplementary file 6 [file Data_Sheet_6.DOCX]

**Supplementary Data S6: Top hit list of SNPs associated to central sensitization in the GWAS cohort**

| Rank | Chr. | SNP ID | Genomic position | Gene/Region | Major allele | Minor allele | MAF (EUR)^a^ | MAF study | P | BETA | CI (95%) | SE | STAT |
| --- | --- | --- | --- | --- | --- | --- | --- | --- | --- | --- | --- | --- | --- |
| 1 | **17** | **rs4796604** | **exonic** | **HAP1** | **A** | **G** | **0.500** | **0.493** | **4.78E-06** | **0.091** | **0.053 - 0.130** | **0.020** | **4.666** |
| *2* | *11* | *rs10834705^b^* | *intergenic* | *LUZP2/ANO3* | *A* | *G* | *0.277* | *0.299* | *1.32E-05* | *0.102* | *0.057 - 0.147* | *0.023* | *4.437* |
| *3* | *13* | *rs2497410^b^* | *intergenic* | *LINC00375/LINC00351* | *C* | *T* | *0.302* | *0.329* | *2.59E-05* | *-0.095* | *-0.138 - -0.51* | *0.022* | *-4.279* |
| *4* | *6* | *rs10807205^b^* | *intronic* | *KCNK17* | *C* | *T* | *0.247* | *0.225* | *2.62E-05* | *-0.106* | *-0.155 - -0.057* | *0.025* | *-4.276* |
| 5 | 1 | rs10889198 | ncRNA_intronic | LOC101926964 | C | T | 0.285 | 0.291 | 3.91E-05 | 0.103 | 0.055 - 0.152 | 0.025 | 4.180 |
| 6 | 8 | rs13270557 | intergenic | MTSS1/MIR4662B | A | G | 0.457 | 0.449 | 4.69E-05 | -0.087 | -0.129 - -0.046 | 0.021 | -4.136 |

Chr. = chromosome

SNP ID = Single nucleotide polymorphism identification number)

MAF = minor allele frequency

P = asymptotic p-value for t-statistic

BETA = regression coefficient

CI 95% = lower and upper limit of 95 % confidence interval

SE = standard error

STAT= t-statistic coefficient

^a^ data for European population from 1000 genome repository (<http://grch37.ensembl.org/>), ^b^ failed sensitivity analysis for comedication
