# Supplementary material for: Uncovering a Genetic Polymorphism Located in Huntingtin Associated Protein 1 in Modulation of Central Pain Sensitization Signaling Pathways
Source: Front Neurosci. 2022 Jun 28;16:807773. doi: 10.3389/fnins.2022.807773 (PMC9274135; doi:10.3389/fnins.2022.807773)
Supplement: Supplementary file 7 [file Data_Sheet_7.DOCX]

**Supplementary Data S7: Differences in NFR threshold between study subgroups**


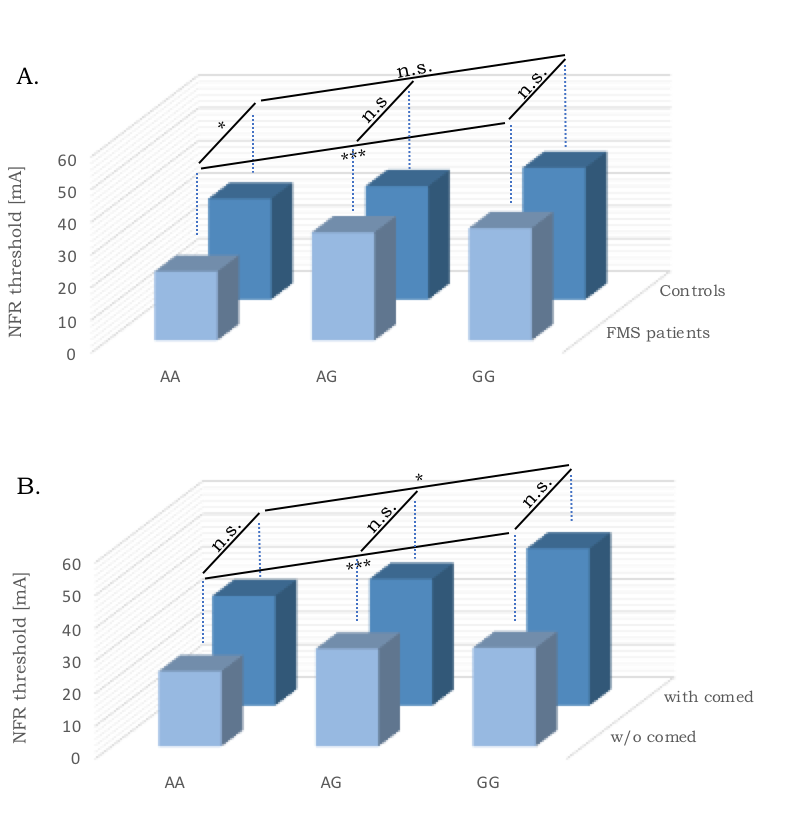


Legend for statistical significance taking into account correction for multiple testing: n.s. = non-significant, * significant, *** highly significant, with cut-offs at *p* < .05 for significant and *p* < .001 for highly significant differences.

Pairwise comparisons are calculated with Mann-Withney and correlations with Kendall statistics.
