# Supplementary material for: Uncovering a Genetic Polymorphism Located in Huntingtin Associated Protein 1 in Modulation of Central Pain Sensitization Signaling Pathways
Source: Front Neurosci. 2022 Jun 28;16:807773. doi: 10.3389/fnins.2022.807773 (PMC9274135; doi:10.3389/fnins.2022.807773)
Supplement: Supplementary file 8 [file Data_Sheet_8.DOCX]

Supplementary material S8: Summary of QST and other clinical scale data

|  | N  (FMS/ Ctrl) | Mean (± sd) | | Comparison  FMS vs control  p-value (W)^a^ | Correlation vs NFR threshold  p-value (Rho)^b^ |
| --- | --- | --- | --- | --- | --- |
|  |  | FMS patients | Controls |  |  |
| NFR (mA) | 284 (212/72) | 34.8 ± 22.4 | 39.9 ± 21.0 | 0.010 (6072) | nd |
| Perception threshold (°C) | |  |  |  |  |
| warmth | 248 (179/69) | 34.5 ± 1.2 | 34.3 ± 0.8 | 0.434 (6572) | 0.076 (0.113) |
| cold | 248 (179/69) | 30.1 ± 1.5 | 29.8 ± 3.5 | 0.518 (5848) | 0.608 (-0.033) |
| Pain threshold (°C) | |  |  |  |  |
| hot | 248 (179/69) | 41.0 ± 4.2 | 43.7 ± 5.3 | **3.45E-06** (3825) | 0.053 (0.123) |
| cold | 248 (179/69) | 19.3 ± 8.8 | 9.4 ± 8.9 | **3.45E-12** (9698) | **0.002** (-0.194) |
| Cold pressor pain tolerance threshold | | |  |  |  |
| time (sec) | 232 (164/68) | 19.7 ± 22.4 | 40.7 ± 33.8 | 1.39E-11 (2432) | 0.027 (0.145) |
| Questionnaires | |  |  |  |  |
| FIQ^c^ | 172 (172/-) | 55.0 ± 15.2 | - | nd | 0.972 (-0.003) |
| PCS | 206 (158/48) | 34.6 ± 7.1 | 55.1 ± 3.2 | **< 2.2e-16** (58) | 0.577 (0.039) |
| PGWB | 242 (180/62) | 51.0 ± 19.5 | 87.0 ± 11.4 | **< 2.2e-16** (663) | 0.031 (0.138) |

^a^ Mann-Withney Mann-Whitney-Wilcoxon test

^b^ Spearman correlation

^c^ The Fibromyalgia Impact Questionnaire (FIQ) was only given to FMS patients.

Corrected p-value threshold = 0.05/8 = 0.006
