# Supplementary material for: Uncovering a Genetic Polymorphism Located in Huntingtin Associated Protein 1 in Modulation of Central Pain Sensitization Signaling Pathways
Source: Front Neurosci. 2022 Jun 28;16:807773. doi: 10.3389/fnins.2022.807773 (PMC9274135; doi:10.3389/fnins.2022.807773)

**Supplementary Data S9: Hap1^K4R^ mutant protein has similar expression levels and distribution as the wild-type protein**

a. Total protein extract from SH-SYS5 cells expressing HAP1^wt^-, HAP1^K4R^-FLAG tagged or the corresponding empty construct, 48h post transfection. b. Subcellular fractionation of SH-SYS5 cells expressing HAP1^wt^-, HAP1^K4R^-FLAG tagged or the corresponding empty construct. CEB: Cytosolic fraction, MEB: Membrane fraction, PEB – Cytoskeletal (or pellet) fraction. Figure panels were cropped for clarity, the corresponding full length gels are presented on the next page.


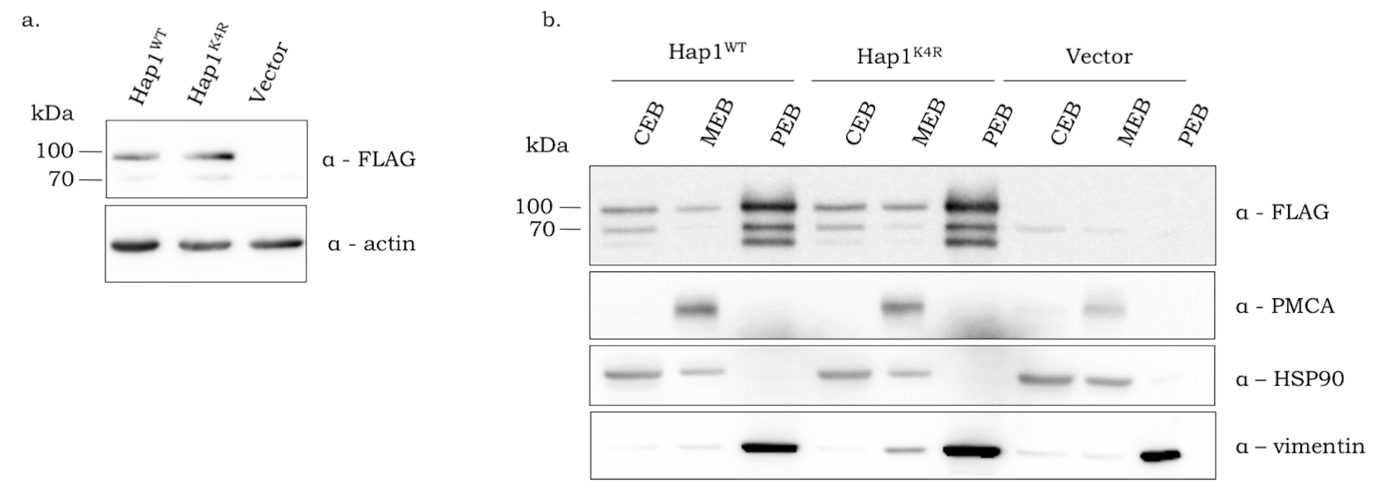


Full sized images:


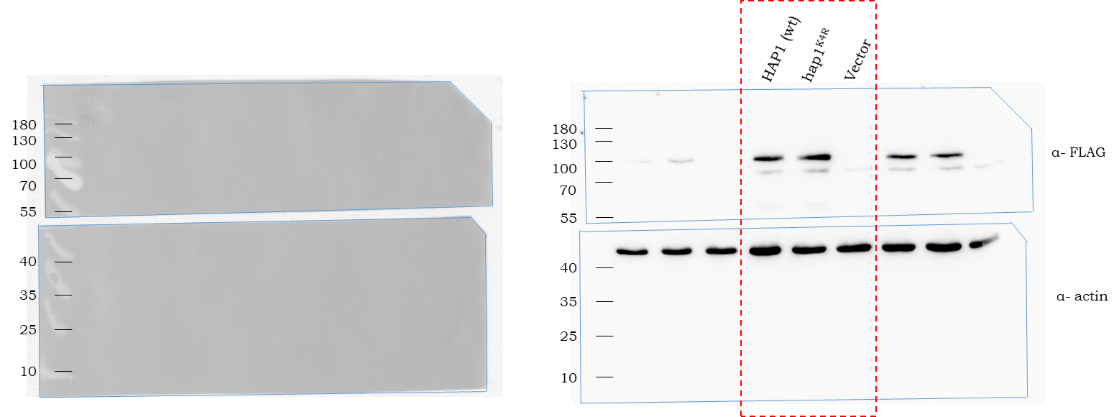


a.

b.


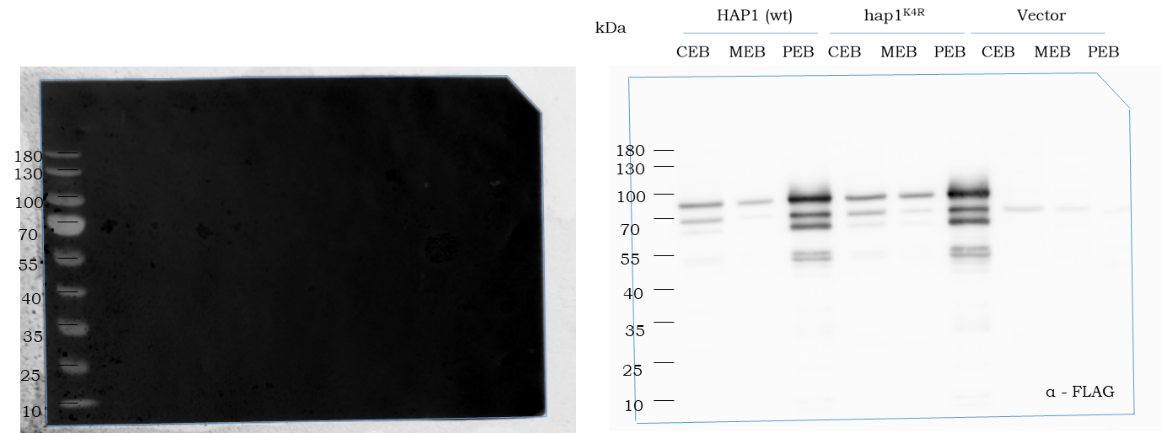


Stripped

membrane


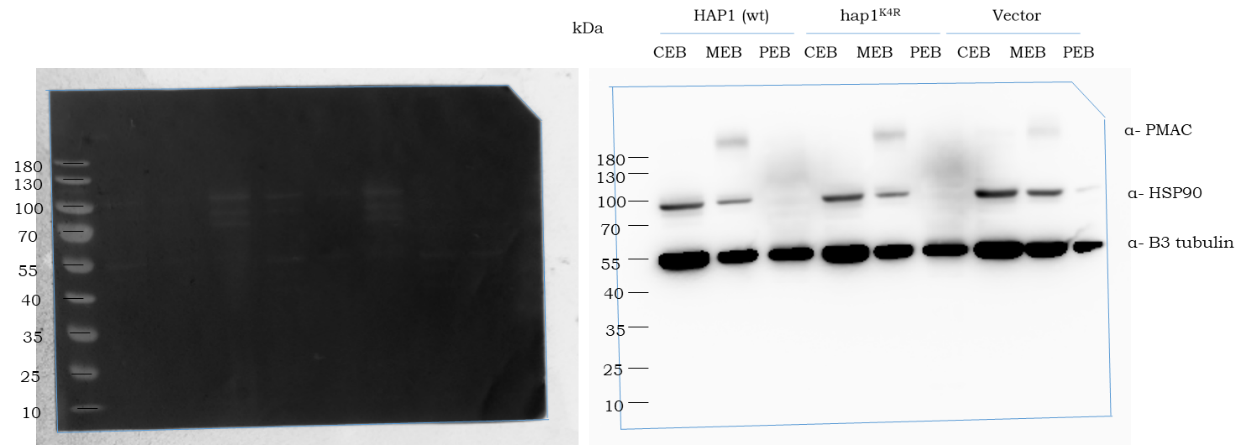


Stripped

membrane


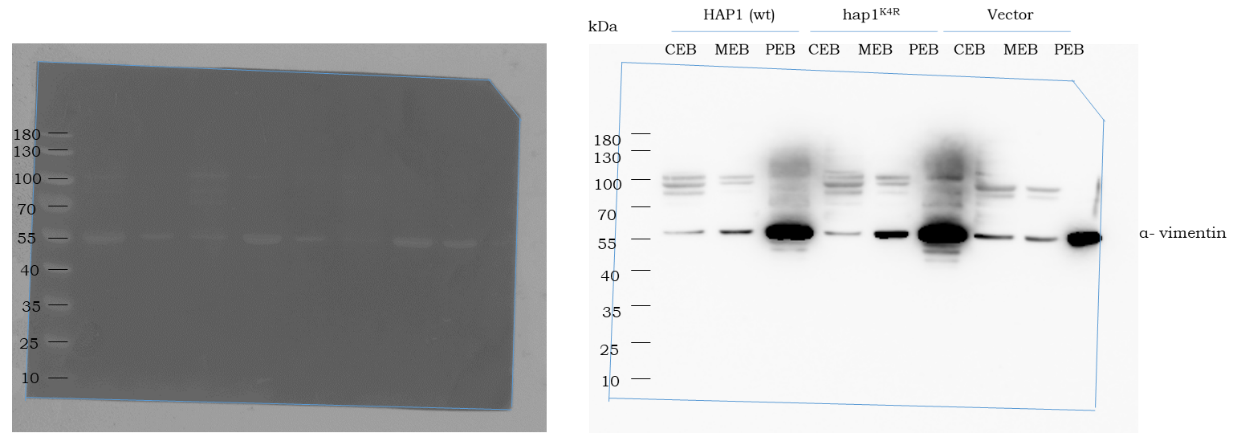

Supplement: Supplementary file 9 [file Data_Sheet_9.DOCX]
