# Supplementary material for: Uncovering a Genetic Polymorphism Located in Huntingtin Associated Protein 1 in Modulation of Central Pain Sensitization Signaling Pathways
Source: Front Neurosci. 2022 Jun 28;16:807773. doi: 10.3389/fnins.2022.807773 (PMC9274135; doi:10.3389/fnins.2022.807773)
Supplement: Supplementary file 10 [file Data_Sheet_10.DOCX]

**Supplementary Data S10: GWAS study power calculations**

Calculation performed according to Gwas-power R package for linear regression models [1].

For N = 300, p-val = 1^E^-5

| qsq | 0.01 | 0.02 | 0.03 | 0.04 | 0.05 | 0.06 | 0.07 | 0.08 | 0.09 | 0.10 |
| --- | --- | --- | --- | --- | --- | --- | --- | --- | --- | --- |
| Power | 0.004 | 0.026 | 0.085 | 0.189 | 0.329 | 0.484 | 0.631 | 0.755 | 0.849 | 0.913 |

N=300


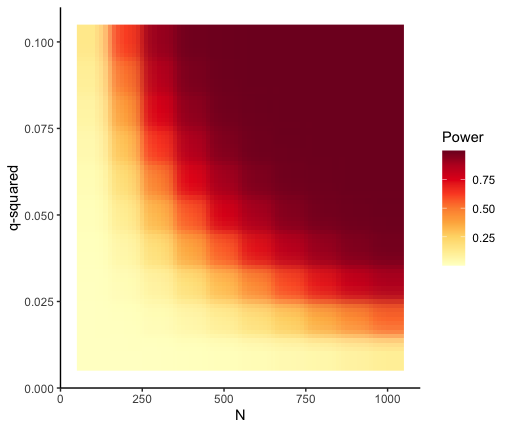

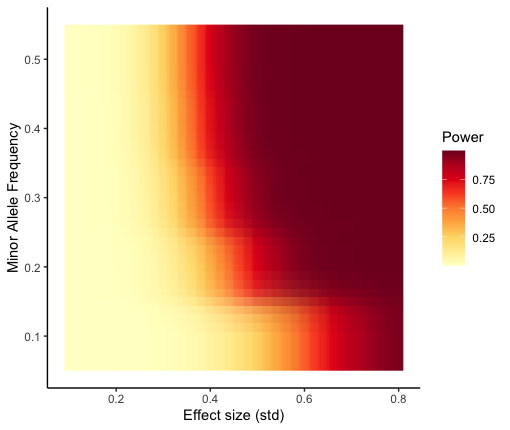


For replication study:

According to the GWAS-Power equations a replication study for rs4796604 in a European population were MAF = 0.5 would require around 200 participants to obtain a p-value of 0.05.

Reference:

[1] Visscher PM, Wray NR, Zhang Q, et al. 10 Years of GWAS Discovery: Biology, Function, and Translation. Am J Hum Genet 2017;101(1):5-22. doi: 10.1016/j.ajhg.2017.06.005.*
